# Supplementary material for: How Progesterone Receptor Expression Impacts Platinum Sensitivity in Ovarian Clear Cell Carcinoma: Insights from Clinical and Experimental Perspectives
Source: Int J Mol Sci. 2024 Jul 20;25(14):7942. doi: 10.3390/ijms25147942 (PMC11276830; doi:10.3390/ijms25147942)

Supplementary Table S1. Univariate Cox regression analyses of progression-free (PFS) and overall survival (OS).

	PFS			OS		
Factors	HR	95% CI	*P* value	HR	95% CI	*P* value
Menopause	0.629	0.305-1.296	0.209	1.801	0.831-3.903	0.136
Parity (≥ 1 vs. 0)	0.725	0.355-1.481	0.378	0.746	0.348-1.601	0.452
Concurrent endometriosis (yes vs. no)	0.087	0.012-0.640	**0.016**	0.112	0.015-0.825	0.032
CA125 (≥ 35 vs. <35)	6.774	0.919-49.95	0.061	7.265	0.979-53.92	0.053
H-score (≥ 50 vs. <50)	0.514	0.156-1.700	0.276	0.491	0.146-1.650	0.250
FIGO stage (III/IV vs. I/II)	5.469	2.657-11.26	**<0.001**	6.226	2.89-13.40	**<0.001**

Statistically significant *p* values are in bold.

Supplementary Figure S1

Positive control from breast cancer tissue. 400X, scale bar 50µm

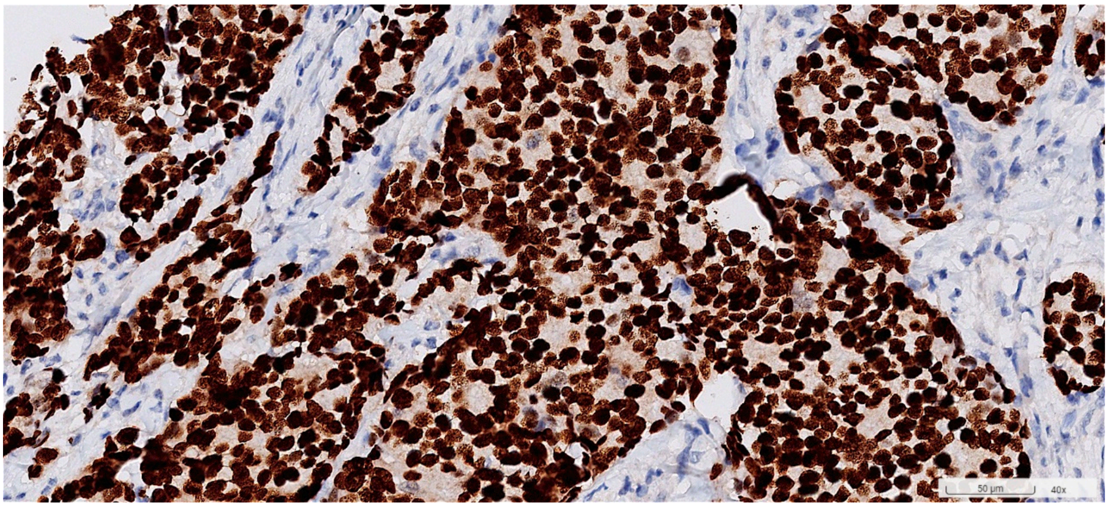

Supplementary Figure S2

A. Kaplan-Meier curves displaying the estimated progression-free survival probability for the strong PR H-score and weak PR H-score groups.

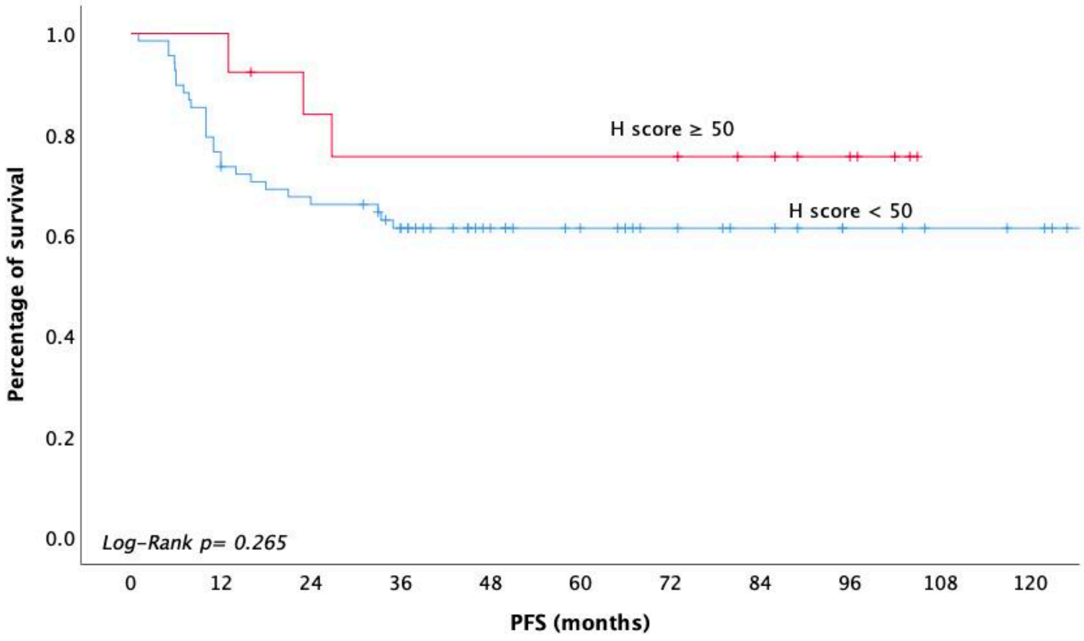

B. Kaplan-Meier curves displaying the estimated overall survival probability for the strong PR H-score and weak PR H-score groups.

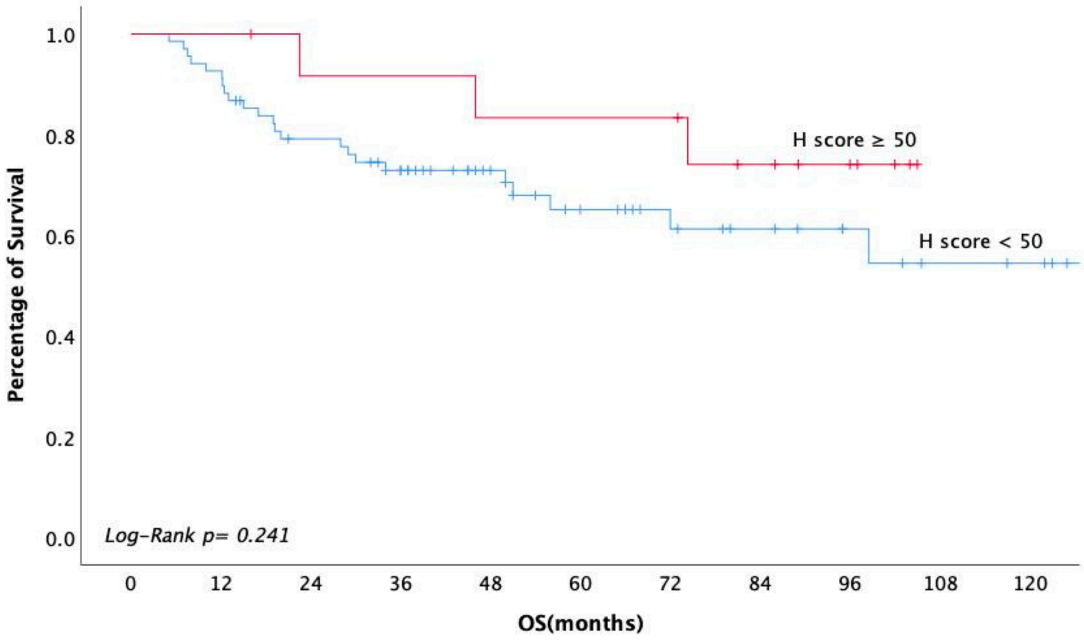

Supplement: Supplementary file 1 [file ijms-25-07942-s001.zip › ijms-3075225-supplementary.pdf]
